# The prognostic value of the combined neutrophil-to-lymphocyte ratio (NLR) and neutrophil-to-platelet ratio (NPR) in sepsis

**DOI:** 10.1038/s41598-024-64469-8

**Published:** 2024-07-02

**Authors:** Yue Zhang, Wang Peng, Xiangrong Zheng

**Affiliations:** 1 Department of Pediatrics, Xiangya Hospital, Central South University, 87 Xiangya Road, Changsha, 410008 Hunan China; 2 The Center of Respiratory Medicine, Xiangya Hospital, Central South University, 87 Xiangya Road, Changsha, 410008 Hunan China

**Keywords:** MIMIC IV, Prognostic value, Neutrophil-to-lymphocyte ratio (NLR), Neutrophil-to-platelet ratio (NPR), Sepsis, Biomarkers, Epidemiology, Experimental models of disease

## Abstract

Sepsis is a severe disease characterized by high mortality rates. Our aim was to develop an early prognostic indicator of adverse outcomes in sepsis, utilizing easily accessible routine blood tests. A retrospective analysis of sepsis patients from the MIMIC-IV database was conducted. We performed univariate and multivariate regression analyses to identify independent risk factors associated with in-hospital mortality within 28 days. Logistic regression was utilized to combine the neutrophil-to-lymphocyte ratio (NLR) and the neutrophil-to-platelet ratio (NPR) into a composite score, denoted as NLR_NPR. We used ROC curves to compare the prognostic performance of the models and Kaplan–Meier survival curves to assess the 28 day survival rate. Subgroup analysis was performed to evaluate the applicability of NLR_NPR in different subpopulations based on specific characteristics. This study included a total of 1263 sepsis patients, of whom 179 died within 28 days of hospitalization, while 1084 survived beyond 28 days. Multivariate regression analysis identified age, respiratory rate, neutrophil-to-lymphocyte ratio (NLR), neutrophil-to-platelet ratio (NPR), hypertension, and sequential organ failure assessment (SOFA) score as independent risk factors for 28 day mortality in septic patients (P < 0.05). Additionally, in the prediction model based on blood cell-related parameters, the combined NLR_NPR score exhibited the highest predictive value for 28 day mortality (AUC = 0.6666), followed by NLR (AUC = 0.6456) and NPR (AUC = 0.6284). Importantly, the performance of the NLR_NPR score was superior to that of the commonly used SOFA score (AUC = 0.5613). Subgroup analysis showed that NLR_NPR remained an independent risk factor for 28 day in-hospital mortality in the subgroups of age, respiratory rate, and SOFA, although not in the hypertension subgroup. The combined use of NLR and NPR from routine blood tests represents a readily available and reliable predictive marker for 28 day mortality in sepsis patients. These results imply that clinicians should prioritize patients with higher NLR_NPR scores for closer monitoring to reduce mortality rates.

## Introduction

Sepsis is a severe infectious disease that remains a significant medical challenge in clinical practice. The Global Burden of Disease Study revealed that in 2017, there were 48.9 million cases of sepsis worldwide, resulting in 11 million deaths, accounting for 19.7% of all global deaths^1^. Despite advances in the early diagnosis and treatment of sepsis, the prognosis for those affected remains unfavorable, with high mortality rates of nearly 41.9%^2^. Therefore, predicting the prognosis of sepsis and implementing timely and effective treatment measures are crucial for improving patient survival and clinical outcomes.

In recent years, increasing evidence has suggested that inflammation and immune response play a pivotal role in the occurrence and progression of sepsis^3^. The neutrophil-to-lymphocyte ratio (NLR), neutrophil-to-platelet ratio (NPR)and neutrophil to monocyte ratio (NMR) are blood-based inflammatory markers, have been widely used to assess patients’ inflammatory status and immune function^4–6^. NLR has been suggested as a prognostic marker for sepsis^7,8^, NPR for infective endocarditis^9^ and myocardial infarction^10^, and NMR for COVID-19^6^. As such, they have attracted attention as potential prognostic indicators to assist in risk assessment and clinical decision-making.

However, current research on the combined application of NLR, NPR and NMR in the prognosis assessment of sepsis is still relatively limited, and further clarification of their importance and clinical utility is needed. Additionally, the prognosis of sepsis is influenced by multiple factors, including patient age, etiology, severity and baseline health status. Therefore, the prognosis assessment of sepsis should consider multiple factors in a comprehensive manner. This study investigates the predictive value of the combined NLR and NPR for the prognosis of sepsis, with the goal of providing more accurate and reliable evidence for prognosis assessment, and personalized treatment of sepsis patients in clinical practice.

## Methods

### Database

The patient data for this study were sourced from the publicly available database established by the Computational Physiology Laboratory at the Massachusetts Institute of Technology (MIMIC-IV v2.2, https://physionet.org/content/mimiciv/2.2/)^11,12^. The data collected encompassed information from all admitted patients at Beth Israel Deaconess Medical Center (BIDMC) from 2008 to 2019, including hospitalization duration, laboratory tests, medication treatments, vital signs, and other relevant data. To protect patient privacy, all personal information was replaced with random codes used to prevent patient identification; therefore, we did not require informed consent or ethical approval from the patients. The first author, Yue Zhang, completed the Collaborative Institutional Training Initiative (CITI) program and passed the exams on “Conflict of Interest” and “Data or Sample Only Research” (ID: 53049076), certifying the research team’s eligibility to use the data.

### Population selection criteria

**Figure 1:**
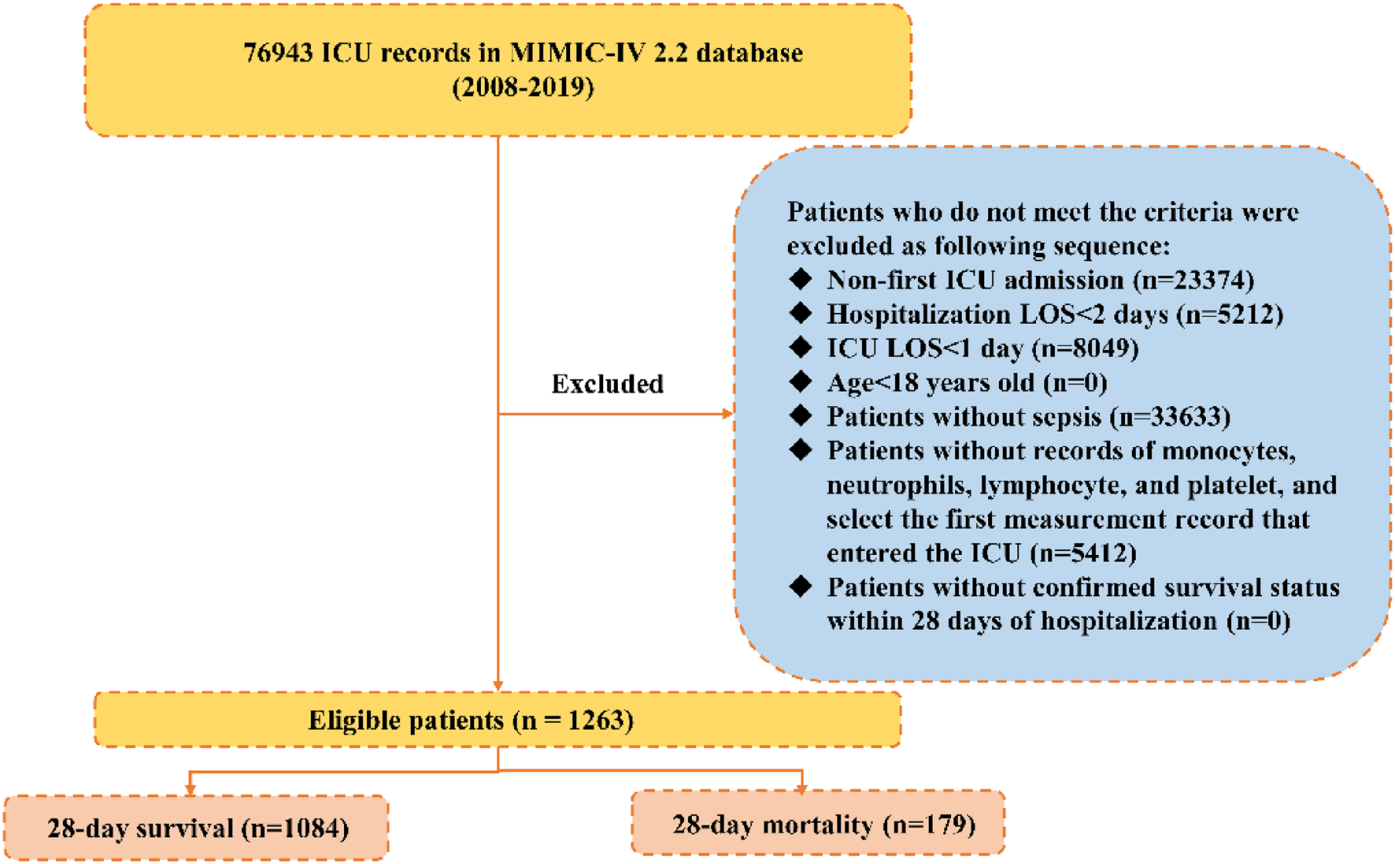
Study flow diagram in the present study. (*MIMIC* Medical Information Mart for Intensive Care).

As illustrated in Fig. 1, a total of 76,943 intensive care unit (ICU) admissions were recorded in the MIMIC-IV database. Sepsis was diagnosed according to the sepsis-3 criteria, wherein patients with documented or suspected infection and an acute change in total Sequential Organ Failure Assessment (SOFA) score of ≥ 2 points were considered to have sepsis^13^. Patients requiring ICU care generally have severe conditions impacting organ function and raising the risk of complications. We recognized that previous ICU admissions could significantly affect patient homogeneity. To reduce selection bias, we included data only from the first ICU admission for patients with multiple ICU stays in the same hospital visit. Due to significant physiological differences between children and adults, patients younger than 18 were excluded. Additionally, to minimize confounding factors associated with short-term hospitalization, patients who spent less than 48 h in the hospital or fewer than 24 h in the ICU were excluded. Furthermore, patients with missing data on key variables (monocytes, neutrophils, lymphocytes, and platelets) and those with missing visits within 28 days were also excluded from the study. Ultimately, 1263 patients were selected for this study and categorized into two groups based on their outcome: survivors (n = 1084) and non-survivors (n = 179).

### Data extraction

We focused on neutrophil-to-lymphocyte ratio (NLR), neutrophil-to-platelet ratio (NPR), and neutrophil-to-monocyte ratio (NMR) as our primary variables of interest. Data on monocytes, neutrophils, lymphocytes, and platelets were obtained from the first routine blood test after admission to minimize the impact of confounding factors. These factors include the participants’ basic characteristics (gender, age, body mass index (BMI)), vital signs (heart rate, respiratory rate, mean blood pressure, peripheral capillary oxygen saturation (SpO2)), laboratory indicators (neutrophil count, lymphocyte count, monocyte count, and platelet count were converted into NLR, NPR, and NMR through ratio transformations), comorbidities (hypertension, diabetes, anemia, coronary artery heart disease, acute kidney failure, chronic kidney disease, heart failure), mechanical ventilation, and Sequential Organ Failure Assessment (SOFA). Logistic regression was utilized to combine the neutrophil-to-lymphocyte ratio (NLR) and the neutrophil-to-platelet ratio (NPR) into a composite score, denoted as NLR_NPR (NLR_NPR = − 2.261 + 3.207 * NPR + 0.019 * NLR). The data extraction was performed using PostgreSQL software (v13.7.1) and PgAdmin software (version 4). All codes for calculating demographic characteristics, laboratory indicators, comorbidities, and severity scores were obtained from the GitHub website.

### Grouping and endpoint events

The primary endpoint of this study was all-cause mortality within 28 days of hospital admission. The 28 day all-cause mortality rate was defined as the ratio of the total number of deaths within 28 days to the total number of patients. The 1263 sepsis patients were divided into the survivor group (n = 1084) and the non-survivor group (n = 179) based on the mortality outcome at 28 days.

### Statistical analysis

All statistical analyses were performed using SPSS software (version 25.0) and Stata 17. Continuous data that followed a normal distribution were presented as mean ± standard deviation (SD), while non-normally distributed variables were presented as median (interquartile range). Categorical data were presented as counts (percentages). Differences between two groups for continuous variables were evaluated using Student’s t-test or Mann–Whitney *U* test, while the chi-squared test was used for comparison of categorical variables. Variance inflation factor and tolerance were used to check for collinearity between dependent variables. Univariate regression analysis was used to identify potential risk factors, and variables with p-values less than 0.1 were included in the multivariate regression analysis to determine independent risk factors for in-hospital mortality. The sensitivity and specificity of NLR, NPR, NLR_NPR, and SOFA scores for predicting 28 day mortality were evaluated through receiver operating characteristic (ROC) analyses. The predictive performance was measured using the area under the curve (AUC), and the significance of differences between models was determined using the DeLong test. The optimal cutoff value for NLR_NPR was determined using the Youden index, and patients were divided into high-value and low-value groups accordingly. The Kaplan–Meier (K-M) method was used to plot unadjusted survival curves, and the log-rank test was used to compare the survival curves between two groups. Subgroup analysis was also conducted to investigate whether NLR_NPR had any impact on different subgroups, including age, respiratory rate, hypertension, chronic kidney disease, and SOFA score.

### Ethical approval

The Massachusetts Institute of Technology (Cambridge, MA) and Beth Israel Deaconess Medical Center (Boston, MA) approved the MIMIC-IV database, and informed consent was obtained for the initial data collection. The study adhered to the principles of the 1964 Helsinki Declaration and its later amendments, or equivalent ethical standards, as well as the ethical standards of institutional and national research committees regarding studies involving human subjects. This study involved analyzing a third-party anonymized publicly available database that had pre-existing institutional review board (IRB) approval.

## Results

### Baseline characteristics and clinical outcomes

A total of 1263 eligible patients from the MIMIC-IV dataset were included in the analysis and categorized into two groups based on their ultimate outcome: survivors (n = 1084) and non-survivors (n = 179). The demographic characteristics and comparative results of these groups are provided in Table 1. Among all patients with sepsis, 1084 (85.8%) survived for longer than 28 days after admission. Compared to the survivor group, patients in the non-survivor group tended to be older (71.93 ± 14.42 vs. 65.29 ± 15.35; P < 0.001) and have higher respiratory rates (20.81 ± 4.16 vs. 19.86 ± 3.92; P = 0.003). In terms of laboratory data, the non-survivor group exhibited significantly elevated levels of NLR (17.04 ± 19.58 vs. 10.56 ± 12.46; P < 0.001), NPR (0.08 ± 0.09 vs. 0.06 ± 0.06; P = 0.001), and NMR (20.56 ± 18.30 vs. 17.46 ± 16.33; P = 0.22) compared to the survivor group. Moreover, patients in the non-survivor group were more likely to have complications such as hypertension and chronic kidney disease compared to the survivor group. Additionally, individuals within the non-survivor group displayed elevated SOFA scores (3 (2–5) vs. 3 (2–4); P = 0.009). No significant differences were observed between the two groups in terms of BMI, heart rate, sex, mean blood pressure, SpO_2_, serum creatinine, lymphocyte count, monocyte count, platelet count, infection location, or ventilation use.

**Table 1:** Baseline characteristics of studied population.

Variables	Total	Survivors	Non-survivors	P-value P
	n = 1263	n = 1084	n = 179	
Male, n (%)	732 (58%)	627 (57.8%)	105 (58.7%)	0.837
Age, years	66.23 ± 15.39	65.29 ± 15.35	71.93 ± 14.42	** < 0.001**
BMI, kg/m^2^	29.15 ± 7.40	29.27 ± 7.48	28.43 ± 6.92	0.158
RR, beats/min	19.99 ± 3.96	19.86 ± 3.92	20.81 ± 4.16	**0.003**
Heart rate, beats/min	86.53 ± 16.78	86.31 ± 16.59	87.87 ± 17.90	0.251
MBP, mmHg	81.50 ± 11.70	81.63 ± 11.85	80.74 ± 10.74	0.346
SpO_2_, (%)	97.10 ± 2.17	97.13 ± 2.06	96.88 ± 2.75	0.148
Laboratory data				
NLR	11.48 ± 13.87	10.56 ± 12.46	17.04 ± 19.58	** < 0.001**
NPR	0.06 ± 0.06	0.06 ± 0.06	0.08 ± 0.09	**0.001**
NMR	17.89 ± 16.65	17.46 ± 16.33	20.56 ± 18.30	**0.022**
Infection location				0.367
Respiratory system	185 (14.6%)	158 (14.6%)	27 (15.1%)	
Blood system	107 (8.5%)	92 (8.5%)	15 (8.4%)	
Urinary system	226 (17.9%)	197 (18.2%)	29 (16.2%)	
Digestive system	29 (2.3%)	26 (2.4%)	3 (1.7%)	
Others	85 (6.7%)	79 (7.3%)	6 (3.4%)	
No record	631 (50%)	532 (49.1%)	99 (55.3%)	
Comorbidities				
Hypertension	331 (17.9%)	299 (27.6%)	32 (17.9%)	**0.006**
Diabetes	139 (11.0%)	123 (11.3%)	16 (8.9%)	0.341
Anemia	86 (6.8%)	74 (6.8%)	12 (6.7%)	0.952
CHD	61 (4.8%)	51 (4.7%)	10(5.6%)	0.611
AKF	266 (21.1%)	229 (21.15%)	37 (20.7%)	0.890
CKD	245 (19.4%)	199 (18.4%)	46 (25.7%)	**0.021**
HF	188 (14.9%)	157 (14.5%)	31 (17.3%)	0.324
Ventilation use, n (%)	1089 (86.2%)	930 (85.8%)	159 (88.8%)	0.276
SOFA	3 (2–4)	3 (2–4)	3 (2–5)	**0.009**

*BMI* body mass index, *RR* respiratory rate, *MBP* mean arterial blood pressure, *SpO*_*2*_ peripheral oxygen saturation, *WBC* white blood rate, *NLR* neutrophil to lymphocyte ratio, *NPR* neutrophil to platelet ratio, *NMR* neutrophil to monocyte ratio, *CHD* coronary heart disease, *AKF* acute kidney failure, *CKD* chronic kidney disease, *HF* heart failure, *SOFA* sequential organ failure assessment.

Significants values are in bold.

### NLR and NPR are independent risk factors for 28 day mortality risk in sepsis patients

Firstly, we conducted a collinearity test on the independent variables within the univariate analysis and found no collinearity among them (Supplementary Table 1). Subsequently, we performed univariate regression analysis to identify the risk factors for 28 day mortality in sepsis patients. The univariate analysis revealed that age (P < 0.001), respiratory rate (P = 0.003), NLR (P < 0.001), NPR (P < 0.001), NMR (P = 0.021), hypertension (P = 0.007), chronic kidney disease (P = 0.022), and SOFA score (P = 0.009) were correlated with 28 day mortality. Details are provided in Table 2**.** Next, we incorporated the potential risk factors identified in the univariate analysis (P < 0.1) into the multivariate regression analyses. The multivariate analysis revealed that age (P < 0.001), respiratory rate (P = 0.012), NLR (P = 0.012), NPR (P = 0.016), hypertension (P = 0.011), and SOFA score (P = 0.048) were significantly associated with 28-day all-cause mortality. Further details can be found in Table 3**.**

**Table 2:** Identification of risk factors for 28 day mortality in septic patients using univariate regression analysis.

Variables	Univariate analysis		
	HR	95% CI	P-valueP
Male, (%)	1.03	0.76–1.38	0.855
Age, years	1.03	1.02–1.04	** < 0.001**
BMI, kg/m^2^	0.98	0.96–1.01	0.155
RR, beats/min	1.05	1.02–1.09	**0.003**
Heart rate, beats/min	1.00	1.00–1.01	0.272
MBP, mmHg	0.99	0.98–1.01	0.342
SpO_2_, (%)	0.95	0.89–1.01	0.113
NLR	1.02	1.01–1.02	** < 0.001**
NPR	1.75	1.45–2.10	** < 0.001**
NMR	1.01	1.00–1.01	**0.021**
Hypertension	0.59	0.40–0.87	**0.007**
CHD	1.16	0.62–2.20	0.640
Diabetes	0.77	0.46–1.29	0.318
Anemia	1.00	0.55–1.79	0.987
AKF	0.97	0.67–1.39	0.859
CKD	1.48	1.09–2.07	**0.022**
HF	1.20	0.81–1.77	0.358
Ventilation use, (%)	1.26	0.79–2.00	0.337
SOFA	1.09	1.02–1.16	**0.009**

NPR values have been log-transformed.

*HR* hazard ratio, *CI* confidence interval, *BMI* body mass index, *RR* respiratory rate, *MBP* mean arterial blood pressure, *SpO*_*2*_ peripheral oxygen saturation, *WBC* white cell blood rate, *NLR* neutrophil to lymphocyte ratio, *NPR* neutrophil to platelet ratio, *NMR* neutrophil to monocyte ratio, *CHD* coronary heart disease, *AKF* acute kidney failure, *CKD* chronic kidney disease, *HF* heart failure, *SOFA* sequential organ failure assessment.

Significants values are in bold.

**Table 3:** Identification of independent risk factors for 28 day mortality in septic patients using multivariate regression analysis.

Variables	Multivariate analysis		
	HR	95% CI	P-value
Age, years	1.03	1.02–1.05	** < 0.001**
RR, beats/min	1.05	1.01–1.09	**0.012**
NLR	1.01	1.00–1.02	**0.012**
NPR	1.55	1.25–1.92	** < 0.001**
NMR	1.00	1.00–1.01	0.249
Hypertension	0.59	0.39–0.89	**0.011**
CKD	1.03	0.72–1.48	0.876
SOFA	1.07	1.00–1.15	**0.048**

NPR values have been log-transformed.

*HR* hazard ratio, *CI* confidence interval, *RR* respiratory rate, *NLR* neutrophil to lymphocyte ratio, *NPR* neutrophil to platelet ratio, *NMR* neutrophil to monocyte ratio, *CKD* chronic kidney disease, *SOFA* sequential organ failure assessment.

Significants values are in bold.

### ROC curve analysis

**Figure 2:**
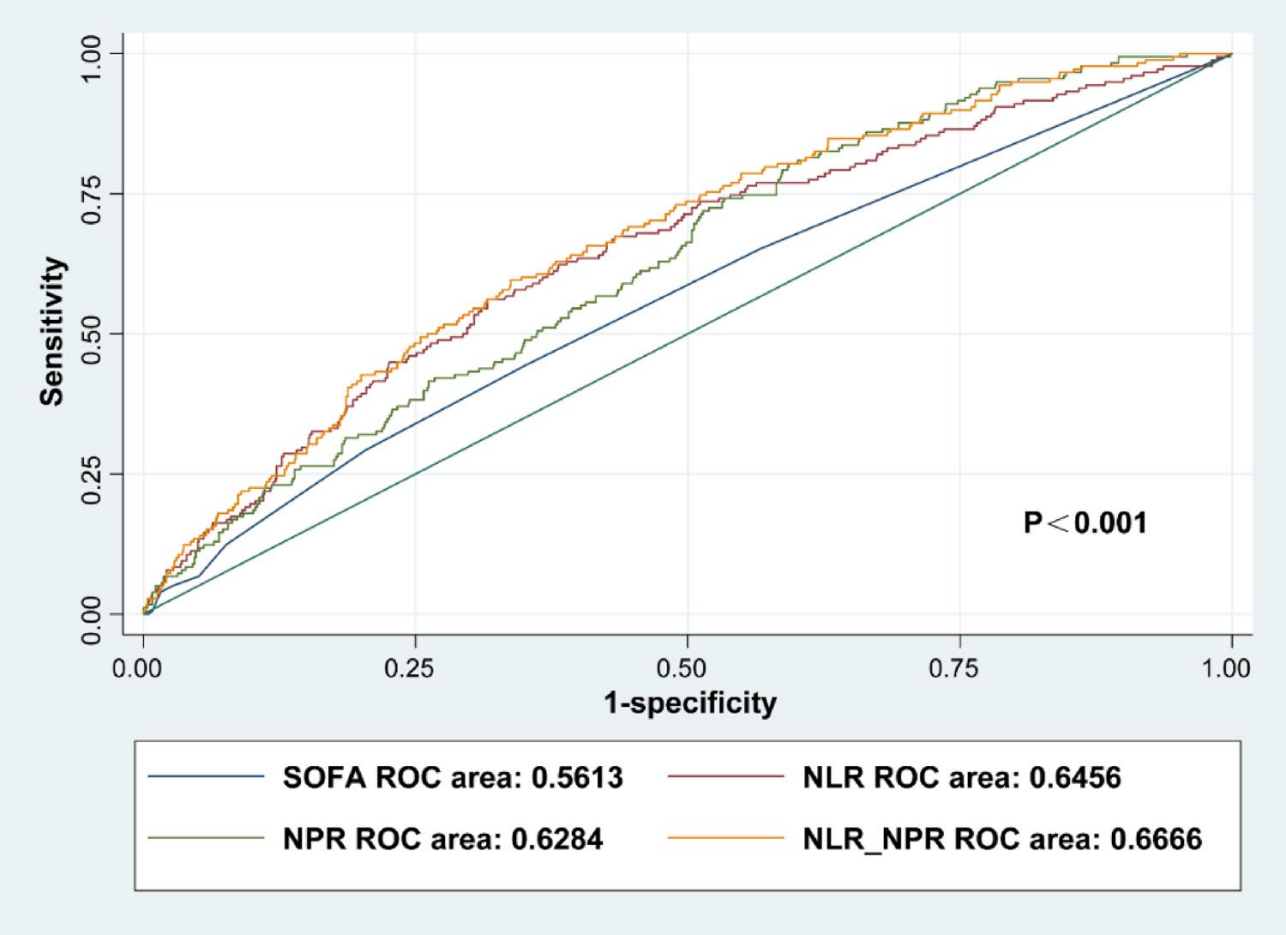
ROC curve for SOFA, NLR, NPR and NLR_NPR.

Subsequently, to achieve a more precise assessment of the predictive value of these variables for 28 day mortality, we performed ROC curve analysis separately for NLR, NPR, the NLR_NPR score, and the SOFA score. Figure 2 illustrates the area under the curve (AUC) of four variables. The NLR_NPR model exhibited the highest AUC (0.667, 95% CI 0.625–0.708), followed by NLR (0.646, 95% CI 0.602–0.690), NPR (0.628, 95% CI 0.587–0.669), and SOFA (0.561, 95% CI 0.515–0.608). To achieve a more precise assessment of the predictive value of NLR_NPR for 28 day mortality, we determined the sensitivity, specificity, cutoff point, and Youden index. The sensitivity and specificity of NLR_NPR at the optimal cutoff value of − 1.89 were 59.6% and 66.2%, respectively, with a Youden index of 0.258.

### Kaplan–Meier curve

**Figure 3:**
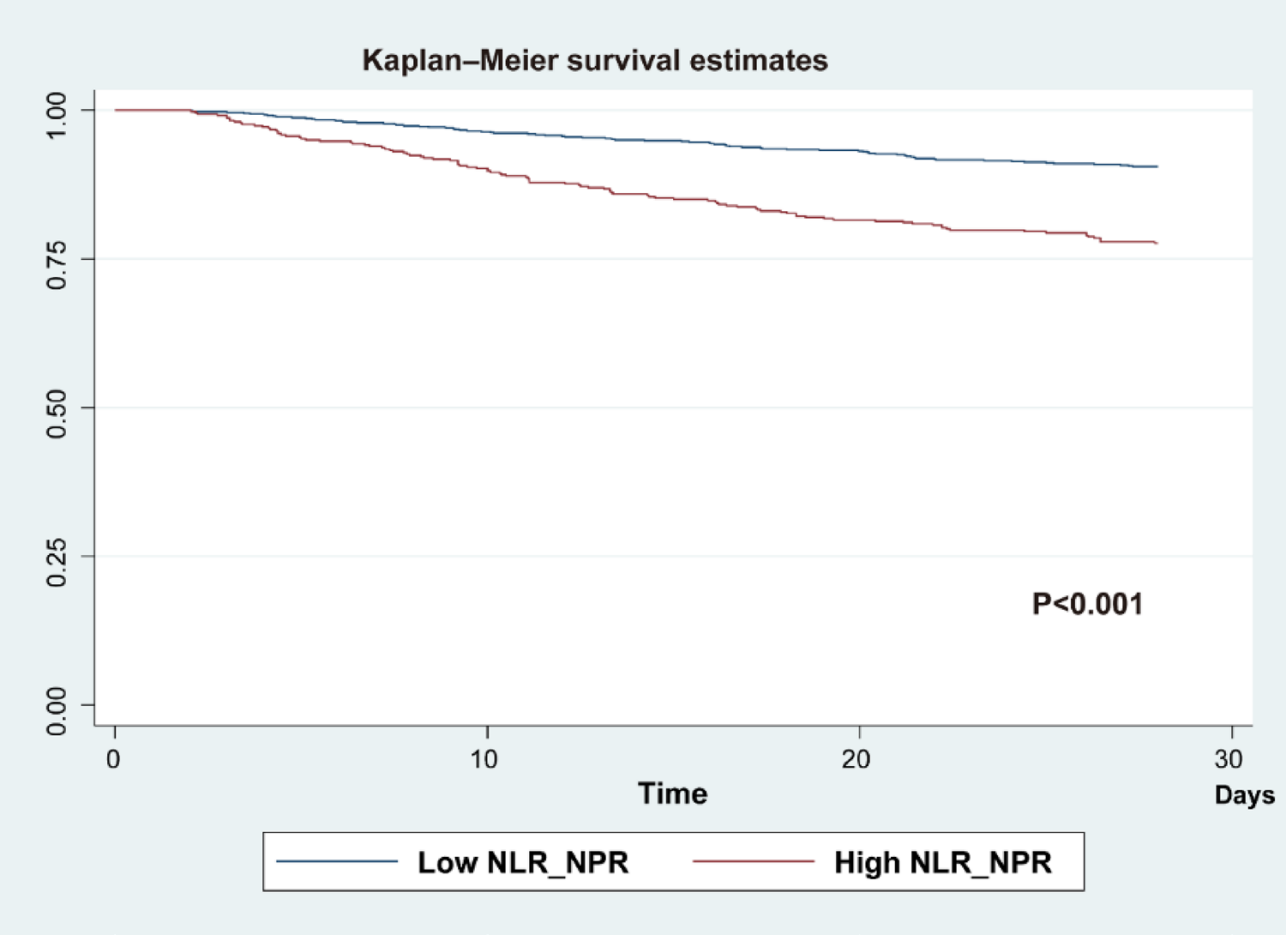
Kaplan–Meier plots for low NLR_NPR group and high NLR_NPR.

Next, based on the optimal cutoff point, patients from the MIMIC-IV dataset were categorized into low NLR_NPR (NLR_NPR < − 1.88, n = 802) and high NLR_NPR (NLR_NPR ≥ − 1.88, n = 461) groups. Kaplan–Meier survival analysis curves were then plotted (Fig. 3), revealing that patients in the high NLR_NPR score group exhibited a significantly higher mortality rate compared to those in the low NLR_NPR score group (P < 0.001).

### Subgroup analysis

**Figure 4:**
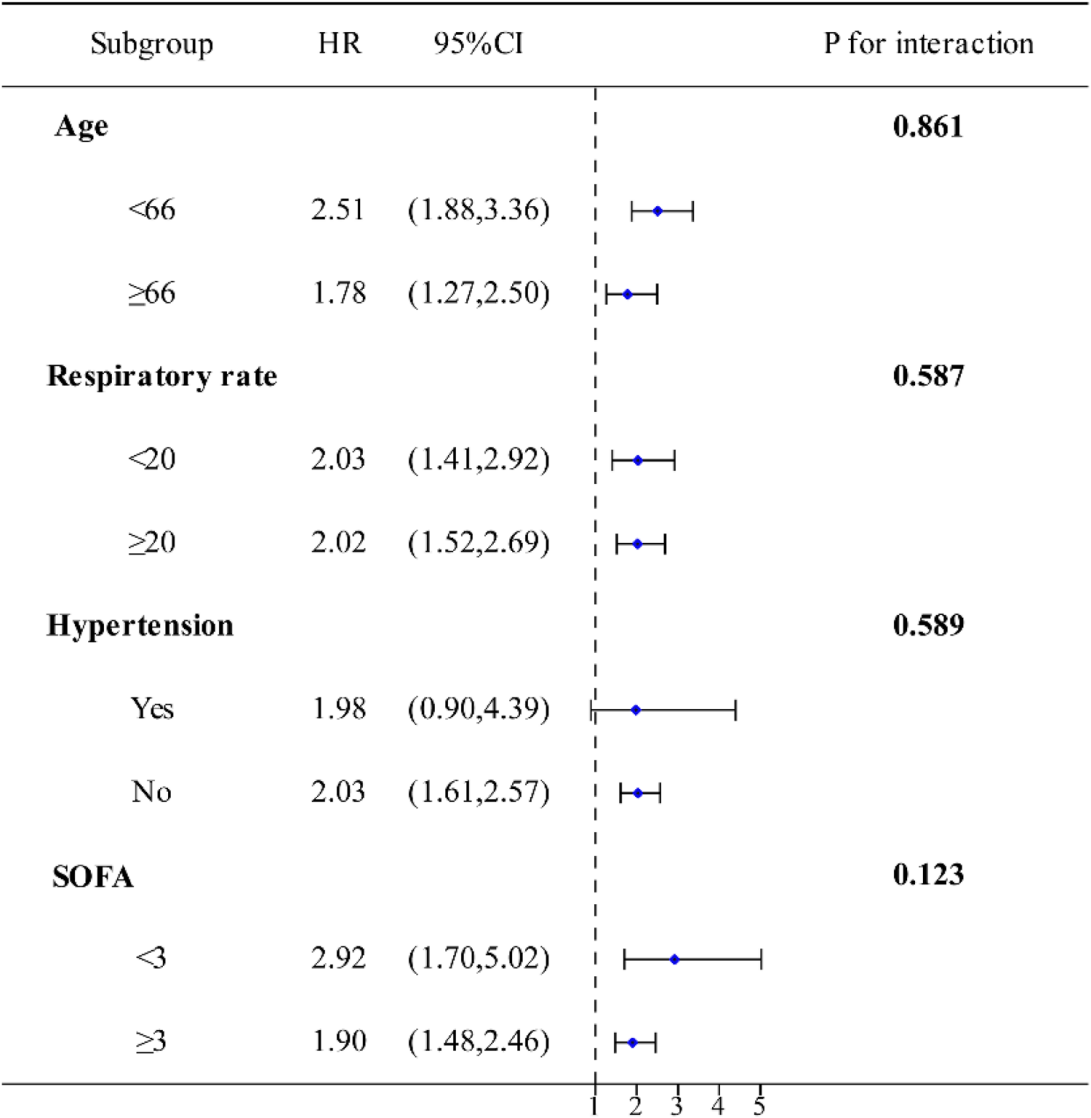
Subgroup analyses. (*HR* hazard ratio, *CI* confidence interval).

Subgroup analysis was conducted based on age, respiratory rate, hypertension, chronic kidney disease, and SOFA score. The forest plot (Fig. 4) revealed no significant interaction between NLR_NPR and any of the subgroups, indicating that NLR_NPR serves as an independent prognostic factor.

## Discussion

In this retrospective study based on a large clinical database, we identified age, respiratory rate, NLR, NPR, hypertension, and SOFA score as being independent risk factors for 28 day mortality in ICU patients with sepsis. Furthermore, we established a prediction model based on routine blood tests and found that the combined NLR and NPR had the highest predictive value (AUC = 0.6666) in terms of predicting mortality within 28 days, followed by NLR (AUC = 0.6456) and NPR (AUC = 0.6284) individually. Notably, the combined NLR and NPR performed better than the commonly used SOFA (AUC = 0.5613). The results of our analysis revealed that the combined NLR and NPR from readily available routine blood tests is a good early monitoring indicator for 28 day mortality in septic patients.

Neutrophils are the first responders to infection and play a crucial role in the elimination of pathogens^14^. However, excessive neutrophil activation can lead to tissue damage and contribute to organ dysfunction in sepsis^15^. Lymphocytes are responsible for regulating the immune response and maintaining immune homeostasis^16^. Lymphocyte co-stimulatory molecules^17^, immune infiltration traits^18^, autophagy^19^ and HLA-associated molecules^20^ collectively influence the inflammatory response in sepsis. A decreased lymphocyte count or function may impair the immune response and increase the risk of sepsis-related complications. Infections trigger the production of cytokines (like granulocyte colony-stimulating factor), accelerating neutrophil production and release, causing a left shift in neutrophil maturation^21^. Concurrently, sepsis induces lymphocyte apoptosis, further disrupting immune balance^22^. This imbalance significantly increases peripheral blood neutrophils while reducing lymphocyte levels, leading to a marked increase in the NLR. Therefore, an increased NLR may reflect an exaggerated pro-inflammatory response and impaired immune regulation, leading to worse outcomes in sepsis. In our study, we observed that patients with higher NLR values had a significantly increased risk of 28 day mortality. These findings are consistent with previous studies that have reported an association between elevated NLR and poor outcomes in septic patients^23–25^. In their retrospective study, Drăgoescu et al. investigated the predictive value of NLR for the occurrence of septic shock in a cohort of 114 septic patients, including 38 patients who developed septic shock. The results showed that NLR had a good sensitivity of 47% and specificity of 78% for predicting the occurrence of septic shock, with an area under the curve (AUC) of 0.631^26^.The study conducted by Liu et al. included 264 patients with sepsis, among whom 78 patients died within 28 days of hospitalization. The researchers found that the AUC of the ROC curve for predicting the outcome of 28 day mortality using NLR was as high as 0.776^27^. An earlier retrospective study using MIMIC III found an AUC value of 0.553 for NLR to predict 28 day mortality in septic patients^28^.In brief, due to differences in study design and sample size, there may be slight variations in the diagnostic performance of NLR in predicting the risk of 28 day mortality in sepsis patients. However, its predictive value remains significant and consistent. Thus, when patients are monitored daily, a reduction in the NLR may serve as an essential indicator of improvement in the inflammatory process.

NPR represents the ratio of neutrophils to platelets in peripheral blood. Sepsis triggers a dysregulated immune response involving inflammation and coagulation pathways^29^. Inflammation and infection can directly activate platelets, leading to platelet aggregation, adhesion to the endothelium, and release of inflammatory mediators and promotes further coagulation activation^30^. Platelets play a key role in the pathogenesis of inflammation-induced coagulation activation^31^. Meanwhile, thrombocytopenia is common in sepsis due to increased platelet consumption in microthrombi, decreased production, and immune destruction^32^. The interaction between neutrophils and platelets involves various mechanisms, including neutrophil-platelet aggregates formation^33^, and platelet activation^34^. Furthermore, platelets support neutrophil extracellular trap (NET) formation, which drives vascular inflammation, coagulation, and immunothrombosis in sepsis^35^.These interactions may contribute to the pro-inflammatory response, endothelial dysfunction, and microvascular thrombosis observed in sepsis. Although there is currently limited research on the association between NPR and sepsis, NPR has been considered a prognostic factor in other inflammatory diseases. For example, it has been used to predict 30 day mortality in patients with ST-segment elevation myocardial infarction undergoing primary percutaneous coronary intervention^10^, as well as in predicting in-hospital and long-term mortality in patients with infective endocarditis^9^.Therefore, NPR, as a marker of the dynamic interplay between neutrophils and platelets, may provide insights into the inflammatory and immune dysregulation that occurs in sepsis.

Monocytes, integral to innate immunity, alongside neutrophils, initiate the inflammatory response^36^. Tissue macrophages (arising from monocytes) are among the first responders, releasing chemokines that recruit neutrophils to the affected site^37^. Several studies have identified that sepsis severity often correlates with reduced monocyte counts, particularly in patients with septic shock. A low monocyte count at admission is considered an independent risk factor for 28 day mortality^38^. Patients with reduced monocyte levels may struggle to contain localized infections, leading to their spread and ultimately resulting in a poorer prognosis. The NMR offers a more comprehensive measure of inflammation severity than individual cell counts. An elevated NMR is recognized as a prognostic marker for poor outcomes in neonatal sepsis^39^. Furthermore, a study on COVID-19 revealed that an NMR of 17.75 or higher is an independent risk factor for in-hospital mortality^6^. In our study, univariate Cox regression analysis showed that NMR was a risk factor for in-hospital mortality within 28 days in sepsis patients. However, after adjusting for potential confounding factors such as age, RR, NLR, NPR, hypertension, and SOFA score, NMR did not show statistically significant association with 28 day mortality(P = 0.249). NMR may be influenced by various factors, such as age, comorbidities, and severity of illness, which may confound the association between NMR and sepsis outcomes. Additionally, the timing of NMR measurement may also impact its association with sepsis outcomes. NMR may fluctuate during sepsis, and a single measurement may not fully capture the dynamic changes in the immune response. Furthermore, the heterogeneity in study design, sample size, and patient populations among different studies may also contribute to the inconsistent findings. Further studies with standardized methodology, larger sample sizes, and well-defined patient populations are needed to better understand the association between NMR and sepsis outcomes.

The clinical implications of our findings are noteworthy. In further analysis, we found that the risk of in-hospital mortality within 28 days was significantly lower in the low NLR_NPR score group compared to the high NLR_NPR score group. Furthermore, in subgroup analyses of age, respiratory rate, and SOFA, NLR_NPR remained an independent risk factor for in-hospital mortality within 28 days, except in the subgroup of patients with hypertension. Although previous studies have not investigated the combined use of NLR and NPR, research on the combined use of NLR, platelet-to-lymphocyte ratio(PLR), LPR, and other routine blood and biochemical markers in diseases such as sepsis^27^, hepatocellular carcinoma^40^ and acute ischemic stroke^41^ has shown that they have a more effective predictive value for disease prognosis compared to single markers. This supports the feasibility of using NLR_NPR to predict adverse prognosis in sepsis and has the potential to aid clinicians in risk assessment, treatment decision-making, and patient management. For example, sepsis patients with an elevated NLR_NPR score may require more aggressive interventions, closer monitoring, and timely escalation of care. Additionally, these biomarkers may be used in the future to identify patients who may benefit from novel therapeutic strategies or targeted interventions aimed at modulating the immune response and improving outcomes in sepsis^42^.

Our study also found that age, respiratory rate, hypertension, and SOFA were independent risk factors for in-hospital mortality within 28 days in patients with sepsis. These findings are consistent with previous research findings from other investigators^43–45^. In the future, the combination of these findings with other established prognostic markers, such as lactate levels, organ dysfunction scores, and biomarkers, may further enhance their predictive accuracy and clinical utility.

It is necessary to acknowledge the limitations of our study. First, our study is retrospective in nature and conducted at a single center, which may limit the generalizability of our findings to other populations or settings. Future multicenter prospective studies with larger sample sizes would help to confirm our findings. Second, while we made efforts to adjust for potential confounding variables, it is essential to acknowledge that complete elimination of residual confounding remains a challenge in our study. Third, our study focused on 28 day mortality as the primary outcome and could be broadened to include other important clinical endpoints, such as ICU admission rate, incidence of complications, length of hospital stays and hospitalization expense.

## Conclusions

In summary, our study found that the combined use of NLR and NPR in a scoring model is an independent risk factor for in-hospital mortality within 28 days for sepsis patients. High NLR_NPR scores indicate an increased risk of death, regardless of age, respiratory rate, or SOFA score, and are effective even in patients without hypertension. This provides an easily accessible and reliable predictive indicator for adverse prognosis in sepsis, suggesting clinicians should prioritize patients with high NLR_NPR scores for closer monitoring to reduce mortality.

### Supplementary Information

Supplementary Table 1.

## Data Availability

Publicly available datasets were analyzed in this study. The datasets presented in the current study are available in the MIMIC IV database (https://physionet.org/content/mimiciv/2.2/).

## References

[1] Rudd KE (2020). Global, regional, and national sepsis incidence and mortality, 1990–2017: Analysis for the global burden of disease study. Lancet.

[2] Fleischmann-Struzek C (2020). Incidence and mortality of hospital- and ICU-treated sepsis: Results from an updated and expanded systematic review and meta-analysis. Intensive Care Med..

[3] van der Poll T, Shankar-Hari M, Wiersinga WJ (2021). The immunology of sepsis. Immunity.

[4] Buonacera A (2022). Neutrophil to lymphocyte ratio: An emerging marker of the relationships between the immune system and diseases. Int. J. Mol. Sci..

[5] Paolisso P (2022). Infarct size, inflammatory burden, and admission hyperglycemia in diabetic patients with acute myocardial infarction treated with SGLT2-inhibitors: A multicenter international registry. Cardiovasc. Diabetol..

[6] Rizo-Tellez SA (2020). The neutrophil-to-monocyte ratio and lymphocyte-to-neutrophil ratio at admission predict in-hospital mortality in mexican patients with severe SARS-CoV-2 infection (Covid-19). Microorganisms.

[7] Salciccioli JD (2015). The association between the neutrophil-to-lymphocyte ratio and mortality in critical illness: An observational cohort study. Crit. Care.

[8] Riche F (2015). Reversal of neutrophil-to-lymphocyte count ratio in early versus late death from septic shock. Crit. Care.

[9] Wei XB (2017). The impact of admission neutrophil-to-platelet ratio on in-hospital and long-term mortality in patients with infective endocarditis. Clin. Chem. Lab. Med..

[10] Somaschini A (2020). Neutrophil to platelet ratio: A novel prognostic biomarker in ST-elevation myocardial infarction patients undergoing primary percutaneous coronary intervention. Eur. J. Prev. Cardiol..

[11] Johnson, A., et al., *MIMIC-IV (version 2.2).* PhysioNet, 2023.

[12] Johnson AEW (2023). MIMIC-IV, a freely accessible electronic health record dataset. Sci. Data.

[13] Esposito S (2017). Sepsis and septic shock: New definitions, new diagnostic and therapeutic approaches. J. Glob. Antimicrob. Resist..

[14] Nauseef WM (2007). How human neutrophils kill and degrade microbes: An integrated view. Immunol. Rev..

[15] Rosales C (2018). Neutrophil: A cell with many roles in inflammation or several cell types?. Front. Physiol..

[16] Geginat J (2013). The CD4-centered universe of human T cell subsets. Semin. Immunol..

[17] Chen Z (2022). Comprehensive characterization of costimulatory molecule gene for diagnosis, prognosis and recognition of immune microenvironment features in sepsis. Clin. Immunol..

[18] Lu J (2022). Characterization of immune-related genes andimmune infiltration features for early diagnosis, prognosis and recognition of immunosuppression in sepsis. Int. Immunopharmacol..

[19] Chen Z (2022). Construction of autophagy-related gene classifier for early diagnosis, prognosis and predicting immune microenvironment features in sepsis by machine learning algorithms. J. Inflamm. Res..

[20] Chen Z (2022). Construction of an HLA classifier for early diagnosis, prognosis, and recognition of immunosuppression in sepsis by multiple transcriptome datasets. Front. Physiol..

[21] Farkas JD (2020). The complete blood count to diagnose septic shock. J. Thorac. Dis..

[22] Zhang Y (2011). Upregulation of programmed death-1 on T cells and programmed death ligand-1 on monocytes in septic shock patients. Crit. Care.

[23] Huang Z (2020). Prognostic value of neutrophil-to-lymphocyte ratio in sepsis: A meta-analysis. Am. J. Emerg. Med..

[24] Zhong X (2021). Neutrophil-to-lymphocyte ratio as a predictive marker for severe pediatric sepsis. Transl. Pediatr..

[25] Li T (2020). Association of neutrophil-lymphocyte ratio and the presence of neonatal sepsis. J. Immunol. Res..

[26] Dragoescu AN (2021). Neutrophil to lymphocyte ratio (NLR)-a useful tool for the prognosis of sepsis in the ICU. Biomedicines.

[27] Liu S (2021). Effects of neutrophil-to-lymphocyte ratio combined with interleukin-6 in predicting 28-day mortality in patients with sepsis. Front. Immunol..

[28] Ye W (2020). The association between neutrophil-to-lymphocyte count ratio and mortality in septic patients: A retrospective analysis of the MIMIC-III database. J. Thorac. Dis..

[29] Tuculeanu G (2023). Coagulation disorders in sepsis and COVID-19-two sides of the same coin? A review of inflammation-coagulation crosstalk in bacterial sepsis and COVID-19. J. Clin. Med..

[30] Iskander KN (2013). Sepsis: Multiple abnormalities, heterogeneous responses, and evolving understanding. Physiol. Rev..

[31] Davis RP, Miller-Dorey S, Jenne CN (2016). Platelets and coagulation in infection. Clin. Transl. Immunol..

[32] Venkata C (2013). Thrombocytopenia in adult patients with sepsis: Incidence, risk factors, and its association with clinical outcome. J. Intensive Care.

[33] Zarbock A, Polanowska-Grabowska RK, Ley K (2007). Platelet-neutrophil-interactions: Linking hemostasis and inflammation. Blood Rev..

[34] von Bruhl ML (2012). Monocytes, neutrophils, and platelets cooperate to initiate and propagate venous thrombosis in mice in vivo. J. Exp. Med..

[35] Dewitte A (2017). Blood platelets and sepsis pathophysiology: A new therapeutic prospect in critically [corrected] ill patients?. Ann. Intensive Care.

[36] Prame Kumar K, Nicholls AJ, Wong CHY (2018). Partners in crime: Neutrophils and monocytes/macrophages in inflammation and disease. Cell Tissue Res..

[37] Grüneboom A (2022). Imaging innate immunity. Immunol. Rev..

[38] Chung H (2019). Circulating monocyte counts and its impact on outcomes in patients with severe sepsis including septic shock. Shock.

[39] Xia X (2022). Elevated neutrophil-to-monocyte ratio as a prognostic marker for poor outcomes in neonatal sepsis. Heliyon.

[40] Schobert IT (2020). Neutrophil-to-lymphocyte and platelet-to-lymphocyte ratios as predictors of tumor response in hepatocellular carcinoma after DEB-TACE. Eur. Radiol..

[41] Gong P (2021). The association of neutrophil to lymphocyte ratio, platelet to lymphocyte ratio, and lymphocyte to monocyte ratio with post-thrombolysis early neurological outcomes in patients with acute ischemic stroke. J. Neuroinflammation.

[42] Wan R (2022). A clinically applicable nomogram for predicting the risk of invasive mechanical ventilation in *Pneumocystis jirovecii* pneumonia. Front. Cell. Infect. Microbiol..

[43] Singer M (2016). The third international consensus definitions for sepsis and septic shock (Sepsis-3). JAMA.

[44] Vincent JL (1996). The SOFA (Sepsis-related organ failure assessment) score to describe organ dysfunction/failure. On behalf of the working group on sepsis-related problems of the European society of intensive care medicine. Intensive Care Med..

[45] Rhodes A (2017). Surviving sepsis campaign: International guidelines for management of sepsis and septic shock: 2016. Intensive Care Med..

