# Supplementary material for: The prognostic value of the combined neutrophil-to-lymphocyte ratio (NLR) and neutrophil-to-platelet ratio (NPR) in sepsis
Source: Sci Rep. 2024 Jul 2;14:15075. doi: 10.1038/s41598-024-64469-8 (PMC11219835; doi:10.1038/s41598-024-64469-8)
Supplement: Supplementary file 1 — Supplementary Table 1. [file 41598_2024_64469_MOESM1_ESM.docx]

**Supplementary table 1** The collinearity analysis between the variables.

|  | **Tolerance**  **RR, beats/min 1.05 1.01-1.09 0.012**  **NLR**  **NLR 1.01 1.00-1.02 0.012**  **NPR 7.19 1.45-36.57 0.016**  **NMR 1 1.00-1.01 0.249**  **Hypertension 0.59 0.39-0.89 0.011**  **CKD 1.03 0.72-1.48 0.876**  **SOFA 1.07 1.00-1.15 0.048** | **VIF** |
| --- | --- | --- |
| Male, (%) | 0.898 | 1.114 |
| Age, years | 0.787 | 1.270 |
| BMI, kg/m^2^ | 0.879 | 1.138 |
| RR, beats/min | 0.784 | 1.275 |
| HR, beats/min | 0.767 | 1.304 |
| MBP, mmHg | 0.893 | 1.120 |
| SpO2, (%) | 0.840 | 1.191 |
| NLR | 0.753 | 1.328 |
| NPR | 0.808 | 1.238 |
| NMR | 0.823 | 1.215 |
| Hypertension | 0.716 | 1.397 |
| CHD | 0.917 | 1.090 |
| Diabetes | 0.891 | 1.123 |
| Anemia | 0.924 | 1.083 |
| AKF | 0.772 | 1.295 |
| CKD | 0.788 | 1.269 |
| HF | 0.789 | 1.267 |
| Ventilation use, (%) | 0.947 | 1.056 |
| SOFA | 0.857 | 1.166 |

**BMI, body mass index; RR, respiratory rate; HR, heart rate; MBP, mean arterial blood pressure; SpO2, peripheral oxygen saturation; WBC, white cell blood rate; NLR, neutrophil to lymphocyte ratio; NPR, neutrophil to platelet ratio; NMR, neutrophil to monocyte ratio; CHD, coronary heart disease; AKF, acute kidney failure; CKD, chronic kidney disease; HF, heart failure; SOFA, sequential organ failure assessment.**
